# Osteopathic Manipulative Treatment Limits Chronic Constipation in a Child with Pitt-Hopkins Syndrome

**DOI:** 10.1155/2017/5437830

**Published:** 2017-01-31

**Authors:** Alessandro Aquino, Mattia Perini, Silvia Cosmai, Silvia Zanon, Viviana Pisa, Carmine Castagna, Stefano Uberti

**Affiliations:** ^1^Research Department, Istituto Superiore di Osteopatia, 20126 Milan, Italy; ^2^Department of Clinical Paediatrics & Obstetrics-Gynaecology, Istituto Superiore di Osteopatia, 20126 Milan, Italy

## Abstract

Pitt-Hopkins Syndrome (PTHS) is a rare genetic disorder caused by insufficient expression of the* TCF4* gene. Children with PTHS typically present with gastrointestinal disorders and early severe chronic constipation is frequently found (75%). Here we describe the case of a PTHS male 10-year-old patient with chronic constipation in whom Osteopathic Manipulative Treatment (OMT) resulted in improved bowel functions, as assessed by the diary, the QPGS-Form A Section C questionnaire, and the Paediatric Bristol Stool Form Scale. The authors suggested that OMT may be a valid tool to improve the defecation frequency and reduce enema administration in PTHS patients.

## 1. Introduction

Pitt-Hopkins Syndrome (PTHS) is a rare genetic disorder characterized by developmental delay (mental retardation), wide mouth, distinctive facial features, and intermittent hyperventilation followed by apnea [1]. The genetic aetiology of PTHS is associated with haploinsufficiency of the* Transcription factor 4 (TCF4)* located on the chromosome 18 (18q21.2). The genetic cause of this disorder was described for the first time in 2007 [1–4], and the overall prevalence of PTHS is unknown. Rosenfeld et al. estimated that the population frequency of PTHS due to 18q21 microdeletion is 1/34,000–1/41,000. The prevalence of PTHS is however clearly underestimated, as many cases are caused by sporadic point mutations [3–5].

Children with PTHS typically present with gastrointestinal disease; particularly constipation is common (75%) and often severe. Only few PTHS patients have comorbidity for Hirschsprung's disease (HSCR) characterized by the absence of enteric ganglia along a variable bowel length (congenital aganglionic megacolon) [1]. In most cases the actual molecular reasons underlying the gastrointestinal features are not identified, even if velocities of the upper gastrointestinal and distal colon transit were impaired in an experimental model of the disease, suggesting that defective regulation by the enteric nervous system (ENS) might be associated with the genetic defect [6]. The regular use of high-fiber diet and/or laxative regimen is recommended [7]. The benefit associated with the use of fibers, such as dietary intervention in children who suffer from constipation, has been demonstrated by two well-designed studies [8, 9]. The North American Society for Paediatric Gastroenterology, Hepatology and Nutrition (NASPGHAN) committee advised osmotic laxatives, magnesium hydroxide, lactulose, lubricant laxative, mineral oil, and Senna [10].

Behavioural modification is an important component of therapy, particularly for children with constipation and encopresis. It involves regular toilet sitting for some minutes a day after meals, combined with a reward system and positive reinforcement from parents [11]. The rarity of PTHS makes it difficult to assess the effects of these approaches. In particular, autistic behaviours and mental retardation, which are typical features of the disease, might restrict the efficacy of these approaches.

Previous studies showed the effectiveness of manual therapies, specially osteopathic treatment, in constipation [12–15]. Osteopathic manipulative therapy, a form of physical manipulation of the body for improvement of the health and body function, has been designated as complementary and alternative medicine (CAM) by the National Institutes of Health (NIH). Osteopathic practitioners use a wide variety of therapeutic manual techniques to improve physiological function and/or support homeostasis that has been altered by somatic (body framework) dysfunctions, that is, an impaired or altered function of related components of the somatic system including skeletal, arthrodial, visceral and myofascial structures and related vascular, lymphatic, and neural elements [16, 17].

In this article, we reported the case of a PTHS patient with chronic constipation, whose bowel functions were improved by Osteopathic Manipulative Treatment (OMT).

## 2. Report of Case

The patient was a Caucasian 10-year-old child with a diagnosis of PTHS genetically confirmed in 2011. The child was born at 38th week (eutocic labour), with a birth weight of 3.620 Kg. The Apgar score was 10 at one and five minutes. No fetal complications during the pregnancy were observed, and the growth was regular throughout the entire period. At birth the child was apparently healthy.

The mother was 44 years old, white Caucasian, at her second pregnancy, nonsmoker, nonalcoholic, and with no history of genetic/congenital disorders. She used no drugs during pregnancy. The father was 54 years old and white Caucasian and had no genetic/congenital disorders. The sister, 16 years old, was born by eutocic delivery and without perinatal problems, with no genetic/congenital disorders. There is no family history of genetic disorders.

The mother reported that constipation had been present for 6 years. The bowel movement frequency varied from 1 to 2 times a week. The child did not seem to have pain during evacuation or in the days between evacuations. The mother always used an enema and abdomen and perineal massage for stimulating child evacuation. The regular use of high-fiber diet and/or laxative regimens were not effective.

The child had its first paediatric osteopathic visit at the Centro di Medicina Osteopatica (Milan) when he was 10 years old. Several features of the disease were present: myopia in his left eye (6.5 dioptres) with use of corrective lenses, strabismus (occlusion bandage 2 hours/day), and dimorphisms (single palmar crease, small penis and hypospadias, and V finger clinodactyly). He had severe bilateral foot hypotonia with planovalgus foot (orthosis), locked knee, dislocated hips, and moderate scoliosis. He presented with PTHS distinctive facial features, episodic hyperventilation, and/or breath-holding while awake. The patient had severe intellectual disability confirmed by child neurologist. Behavioural features comprise happy disposition, stereotypic head and hand movements, sleep disorders, self-aggression, and anxiety. He did not present seizures, but the EEG showed slow theta bands with epileptogenic signs in his temporal regions. The patient used no antiepileptic drugs. Moreover, the patient showed motor development delay and no verbal communication. No surgical history, previous medical treatments besides laxatives, or allergies were documented. The child has never been subjected to colonoscopy or the histologic examination of the rectal tissue.

The child has moderate to severe cognitive delay and an undifferentiated approach to objects (beat, crush). Affective and relational context presents a slightly modulated expression. Communication is limited to very few and limited understanding required rituals. Organization of space and time and attention are very poor with primary autonomies not achieved. The mother has not changed evacuative habits (time and frequency toilet) during the study. The child underwent physiotherapy and swimming once a week and psychomotor activity twice a week and continued with these activities throughout the duration of the study.

## 3. Physical and Osteopathic Evaluation

The patient was alert and oriented and did not appear in distress upon examination. Weight was 30 Kg and height was 130 cm. Vital signs included blood pressure 110–75 mmHg, heart rate 70 bpm, and respiratory rate 15 apm. The abdomen was not tender. No hepatosplenomegaly was found.

The osteopathic evaluation was performed by two osteopathic practitioners with more than 5 years of paediatric clinical experience. The osteopathic structural examination showed bilateral hypertonia of the hamstring and paraspinal muscles, severe ankle muscle hypotonia, fascial diaphragm restriction, anterior right-rotated iliac bone, right-rotated sacrum, left sacroiliac (SI) joint hypomobility, overloaded breathing accessory muscles, and lower chest stiffness. The specific segmental diagnosis was not possible due to lack of patient compliance.

## 4. Osteopathic Manipulative Treatment and Daily Parallel Plan

The patient underwent 6 weekly OMT sessions (session length 30′). During the first two sessions, myofascial-release treatment (MFR) was applied to the abdomen region for abdominal bloating, and strain-counterstrain techniques were applied to the ileocaecal valve and gastroduodenal junction. Furthermore, manipulation of the (ascending, transverse, and descending) colon and mesenteric lift were used to improve colon function, as suggested by Brugman et al. [12]. Additionally MFR, soft-tissue (ST), and articulatory treatment (ART) were used on the pelvic, lumbar, and dorsal spine, relying mostly on passive mobilization techniques for the thoracolumbar trait.

In the following sessions, a diaphragmatic approach was used to treat the thoracic and pelvic diaphragms. Specific neurofascial techniques (rib-raising and suboccipital release) were used.

OMT was consistently well tolerated and adverse effects were not identified. High-fiber and fruits diet, fixed time for evacuations (e.g., 8 pm), massage, and stimulation of his abdomen and perineal area were not modified, throughout the treatment.

## 5. Measurement Tools

During this study three different measurement tools were used:

- A constipation diary (date, hour, effort, urgency, use of laxative, and/or enema),
- QPGS-Form A Section C questionnaire [18],
- Bristol Stool Form Scale (paediatric score) [19].

These tools were administered to the mother to monitor the constipation in the 3 weeks before the first treatment, during the treatment period and during the two follow-up periods both of 5 weeks.

## 6. Bowel Movement Frequency and Enema Administration Frequency

**Figure 1:**
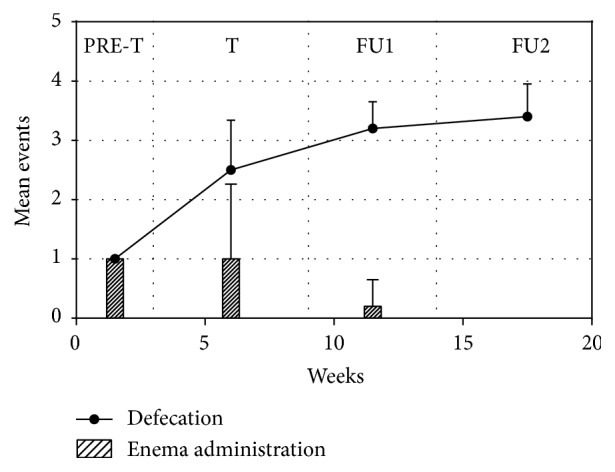
Frequency of defecations and enema administrations per week during the study. PRE-T: 3 pretreatment weeks; T: 6 treatment weeks; FU1: 5 follow-up weeks; FU2: 10 follow-up weeks.

Throughout the 3 weeks before the first treatment, the defecation frequency was 1.0 ± 0.0 per week with a strict association with the enema administration. During the 6-week treatment, the mean number of defecations was 2.5 ± 0.8 per week and enema administration was reduced to 1 ± 1.3 per week. During the follow-up period, the mean evacuation number reached initially 3.2 ± 0.4 per week and enema administration frequency was reduced to 0.2 ± 0.4 (specify the length of the early follow-up period). Later on, the mean evacuation number reached 3.4 ± 0.5 per week, and no enema was administrated (Figure 1).

## 7. QPGS-Form A Section C Questionnaire and Bristol Stool Scale–Paediatric

The Questionnaire on Pediatric Gastrointestinal Symptoms-Rome III Version (QPGS-RIII) is an adaptation and abbreviation of the Questionnaire on Pediatric Gastrointestinal Symptoms (QPGS) that was developed with the support of a grant from the Rome Foundation and that has undergone preliminary validation [18]. The original QPGS assesses the Rome II symptom criteria for paediatric functional gastrointestinal disorders and additional gastrointestinal symptoms. The parent-report version of the QPGS-RIII is suitable for use by parents of children four years of age and older. The questionnaire uses 5-point scales to measure frequency, severity, and duration of symptoms. In addition, it may be scored to assess whether a patient meets the criteria for each of the individual functional gastrointestinal disorders. The QPGS-RIII cannot substitute for the medical evaluation and clinical judgment required for an accurate diagnosis.

The results by a descriptive analysis using the QPGS-Form A Section C questionnaire revealed that items 3, 11, 12, 19, and 20 were modified after the OMT treatment. In detail, weekly defecation frequency (from 1-2 times/week to 3–6 times/week) was improved (item 3). Strain was reduced (from “always” to “sometimes,” item 11); mucus during evacuation was reduced (from “sometimes” to “never,” item 12). Evacuation occurred during the night (item 19, from “no” to “yes”) and complete faecal continence was achieved (item 20). The colonic transit time, as assessed by the Bristol Stool Scale–Paediatric, was not modified.

## 8. Discussion

The etiology of constipation in Pitt-Hopkins Syndrome is unknown. Thus dietary indications (e.g., fibers) and constant use of laxatives and stool softeners are used to handle constipation [12–15, 18–20]. Not surprisingly, this approach often is not effective [21, 22].

Recent evidence suggests that neurological disorders involve a part of the Autonomic Nervous System (ANS), namely, the ENS [23, 24]. The ENS influences the Central Nervous System (CNS) and* vice versa* [25, 26]. The interplay between the two systems is pivotal for the proper bowel functions, including the bowel motility, secretion/absorption, and local blood flow [25, 27]. This is supported by the consideration that the 90% of vagal fibers are afferent in the gut-brain axis [28]. A role of the ENS in neurological disorders, as a trigger or a player, has been advocated [23]. The pathophysiological processes that underlie CNS disease often have enteric manifestations [25]. In a recent study on a murine model of PTHS, the authors provided the first evidence that* TCF4* haploinsufficient mice had a reduced upper gastrointestinal and distal colonic transit velocities [6]. TCF4 is expressed in both glial cells and neurons of the CNS [29] and the differentiation and migration of a subset of neuronal progenitors might be influenced [30]. This suggests that TCF4 expression during the formation of ENS might be required for effective bowel motility [2].

Based on the hypothesis that the genetic defect could cause a specific defect in the regulation of the ENS action we used OMT to restore the ANS balance, the visceral mobility and respiratory function, and the improvement of venous and lymphatic circulatory systems. In particular, we focused on both parasympathetic and sympathetic ANS components, considering that the hyperactivity of ANS leads to decreased peristalsis as well as increased sphincter tone [24]. To influence the Parasympathetic Nervous System (PNS) function, we acted on the vagus nerve by treating the cranial and upper cervical spine. We acted on the II-III and IV sacral roots by approaching the sacrum, the innominate, and pelvic floor thus balancing the external anal sphincter tone [24]. The T6–T12 tract that is correlated with the upper gastrointestinal function and the T12–L2 tract that is correlated with the left half of the colon were manipulated by using MFR, ST, and ART. This approach solved the segmental somatic dysfunctions, maybe reducing indirectly also the sympathetic response via paraspinal muscle inhibition [24].

Furthermore, Spinal Manipulation (SM) could have had a twofold effect, acting on (i) the skeletal muscle hypertonia,* via* the efferent projection from the CNS; (ii) the enteric, sympathetic, and parasympathetic systems,* via* the afferent fibers located in dorsal root or cranial nerve ganglia [24]. The rib-raising technique was carried out in order to decrease the sympathetic ANS activity [31].

Similarly, to what has been described in the experimental model of the disease [6], we observed fascial tension in the upper gastrointestinal and distal colonic traits. The visceral approach was aimed to reduce the fascial restriction. In particular, we applied a specific technique to the ileocaecal valve and gastroduodenal junction. The colon and mesenteric root were manipulated by a lift technique. The visceral approach may have improved constipation by presumably increasing the organ mobility [32], positively changing the peripheral neuroplasticity [33], and implementing the communication between the brain and gut. MFR and STM on the thoracic spine and ribcage together with the treatment of the thoracic diaphragm could have improved the blood and lymphatic bowel vasculature [34]. Also, the approach on the diaphragm might act on the colonic flexures and intra-abdominal pressures [12, 34]. The lymphatic pump treatment might have reduced the enteric inflammation, possible in PTHS patients due to altered WNT/b-catenin/TCF pathway [34, 35].

So far, there are no specific case reports or studies on the use of OMT in PTHS patients with chronic constipation. The pilot studies and case studies [12, 14] that studied specific syndromes did not deal with this disorder. The use of a constipation diary allowed a thorough analysis of changes in the weekly frequency of evacuation.

Limitation of the study includes the limited collaboration of the patient that restricted the possibility of relying on a proper technique procedure and the lack of information on the histological features of the gut mucosa.

The PTHS is an extremely rare syndrome and multidisciplinary reference centers are lacking. Even if clinical experience is limited we feel that results are promising, suggesting that OMT may be beneficial for children affected and suffering from constipation.

The standard medical therapy appears in children to have limited efficacy effect in the medium and long term and has substantial potential side effects [36]. In contrast OMT could represent a long-term effective, noninvasive, and well-tolerated therapy.

Constipation is a common disorder in several neurological disorders, possibly as a consequence of the interrelationship between CNS and ENS [23]. OMT might also have a role in other neurological disorders. Further studies are necessary to verify this hypothesis.

## 9. Conclusions

To our knowledge, this is the first case report that describes the effects of OMT on severe chronic constipation in a PTHS paediatric patient. OMT can be a valid tool to improve the defecation frequency and reduce enema administration, thus improving the quality of life of the PTHS paediatric patients and of their families.
